# Fast Coincidence Filter for Silicon Photomultiplier Dark Count Rate Rejection

**DOI:** 10.3390/s24072084

**Published:** 2024-03-25

**Authors:** Diego Real, David Calvo, Juan de Dios Zornoza, Mario Manzaneda, Rebecca Gozzini, Carlos Ricolfe-Viala, Rafael Lajara, Francisco Albiol

**Affiliations:** 1 IFIC—Instituto de Física Corpuscular, CSIC—Universitat de València, c/Catedrático José Beltrán, 2, 46980 Paterna, Spain; dacaldia@ific.uv.es (D.C.); zornoza@ific.uv.es (J.d.D.Z.); mangarma@alumni.uv.es (M.M.); sara.gozzini@ific.uv.es (R.G.); kiko.albiol@ific.uv.es (F.A.); 2 Automatic Control and Industrial Informatics Institute, Universitat Politècnica de València, Camino de Vera s/n, 46022 Valencia, Spain; cricolfe@ai2.upv.es; 3 Departamento de Ingenieria Electrónica, ETSE—Universitat de Valencia—Burjassot, 46100 Valencia, Spain; jose.r.lajara@uv.es

**Keywords:** time-to-digital converters, neutrino telescopes, silicon photomultipliers, dark noise rate filtering

## Abstract

Silicon Photomultipliers find applications across various fields. One potential Silicon Photomultiplier application domain is neutrino telescopes, where they may enhance the angular resolution. However, the elevated dark count rate associated with Silicon Photomultipliers represents a significant challenge to their widespread utilization. To address this issue, it is proposed to use Silicon Photomultipliers and Photomultiplier Tubes together. The Photomultiplier Tube signals serve as a trigger to mitigate the dark count rate, thereby preventing undue saturation of the available bandwidth. This paper presents an investigation into a fast and resource-efficient method for filtering the Silicon Photomultiplier dark count rate. A low-resource and fast coincident filter has been developed, which removes the Silicon Photomultiplier dark count rate by using as a trigger the Photomultiplier Tube input signals. The architecture of the coincidence filter, together with the first results obtained, which validate the effectiveness of this method, is presented.

## 1. Introduction

Neutrino astronomy has entered a fruitful era with the success of neutrino telescopes. The IceCube Telescope [1] has confirmed the presence of high-energy cosmic neutrinos, correlated a high-energy neutrino with a transient source [2], and has even detected neutrinos from a steady source, NCG 1068 [3]. KM3NeT [4], currently under construction, will focus on finding cosmic neutrino sources in the new multi-messenger era, potentially solving the mystery of high-energy cosmic rays’ origin. This places KM3NeT alongside IceCube at the forefront of many exciting physics topics. Improving the angular resolution of neutrino telescopes is crucial for pinpointing steady point-like sources [5]. The use of Silicon Photomultipliers (SiPMs) [6] in the next generation of telescopes, either alone or in combination with Photomultiplier Tubes (PMTs), will greatly enhance the angular resolution [7]. In neutrino telescopes, an improvement of about 40% in the angular resolution could be reached in seawater by the use of a hybrid detection node composed of SiPMs and PMTs, while, in ice, the improvement could be around 20% because of the higher light scattering. This increases the likelihood of detecting steady point-like sources. SiPMs offer rapid responses and low jitter, crucial for improving angular resolution. The rise time of SiPM pulses is about a quarter of a nanosecond. The transit spread time of SiPM signals has a similar value, which significantly improves the PMTs; in the case of KM3NeT, the PMTs operate at over one nanosecond [8]. Additionally, ice telescopes could benefit from the advantages of SiPMs, including higher photon efficiency and larger detection area. Nevertheless, equipping neutrino telescopes with SiPMs poses a technological challenge, particularly in dealing with their high dark count rate (DCR). After pulses, delayed signals generated by SiPMs can also affect system performance. A technique to mitigate these effects, based on coincidences, is presented in this work. The use of coincidences to decrease the DCR noise has already been proposed in some space missions, such as NUSES, where it has been implemented to decrease the DCR in Analog-to-Digital Converter (ADC) channels reading two SiPMs [9]. In this development the trigger implemented after the ASIC ADC. Additionally, the use of a coincidence filter to remove the DCR by developing coincidences in a SPAD array has also been proposed (SPAD-imagers) [10,11]. The DCR is decreased; however, it requires implementation at the silicon level, and, moreover, it works with at least two coincident photons, therefore filtering single-photoelectron information. Additional, similar performances could be achieved by raising the detection threshold over the single-photoelectron level, which is possible to use in the case of SiPMs without the coincidence implementation in the silicon. The proposal presented in this work is implemented before the TDC acquisition channels, allows the use of standard SiPMs, uses a PMT signal as a trigger, and allows the acquisition of single-photoelectron information in the SiPM channels whenever it is coincident with the triggering PMT channels. The work presented is a feasibility study implemented in ideal conditions and not operated with actual sensors, SiPMs, or PMTs; thus, the actual loss of PDE or the DCR reduction may vary in real conditions. Nevertheless, as the efficiency of the PMTs is lower than that of the SiPMs, a decrease in the detection of single-photoelectron events in the SiPMs is expected unless the detection area of the SiPM is lower than half of the PMT area (to compensate the additional efficiency of the SiPMs), which will be the case in neutrino telescope applications.

This work begins by discussing the advantages and disadvantages of using SiPMs in neutrino telescopes in Section 2. In Section 3, the problems of SiPM DCR are discussed. The DCR filter proposed in this work is presented in Section 4, while the implementation of the filter is discussed in Section 5. The first results are presented in Section 6, followed by the conclusions in Section 7.

## 2. Silicon Photomultipliers in Neutrino Telescopes: Advantages and Disadvantages

SiPMs consist of a Geiger-mode avalanche photodiode array on a common silicon substrate [12], typically housing 1000 microcells (pixels) in a 1 mm^2^ area. Each pixel behaves akin to a photodiode and a quenching resistor in series, ensuring a uniform response. With rise times under a quarter of a nanosecond [6,13] and time transit spreads (TTSs) of similar values [6,14], SiPMs are well suited for the next generation of neutrino telescopes. Using SiPMs in neutrino telescope acquisition nodes, whether in lieu of or alongside PMTs, can significantly enhance angular resolution [7], improving the precision of neutrino telescope readings [5]. Nevertheless, the acquisition electronics requirements go beyond the traditional 1 ns resolution [15]. Beyond their precise timing, SiPMs offer the following various advantages for neutrino telescopes:

- Sensitivity spanning from ultraviolet to near-infrared, ideal for Cherenkov light;
- No need for a high-voltage supply exceeding 100 V, yet comparable gains to traditional PMTs (10^5^–10^6^) are achieved;
- Immunity to electromagnetic fields;
- The SiPMs can be packed, making it possible to make adjustable expansions at a relatively lower cost compared to PMTs [16];
- Excellent single-photon resolution [17];
- Mechanically more robust than PMTs;
- Resilience to stray light due to solid-state technology;
- High photon detection efficiency (PDE), exceeding 50% in blue [18], compared to 30% in KM3NeT PMTs [8];
- Abundance of producers in this growing market with associated R&D [19,20].

The characteristics of SiPMs lead to improved efficiency, detection area, directional sensitivity, and overall angular resolution. On the other hand, the main disadvantage of SiPMs for their use in neutrino telescopes is their extremely high DCR.

## 3. Silicon Photomultiplier Dark Count Rate Challenge

**Figure 1:**
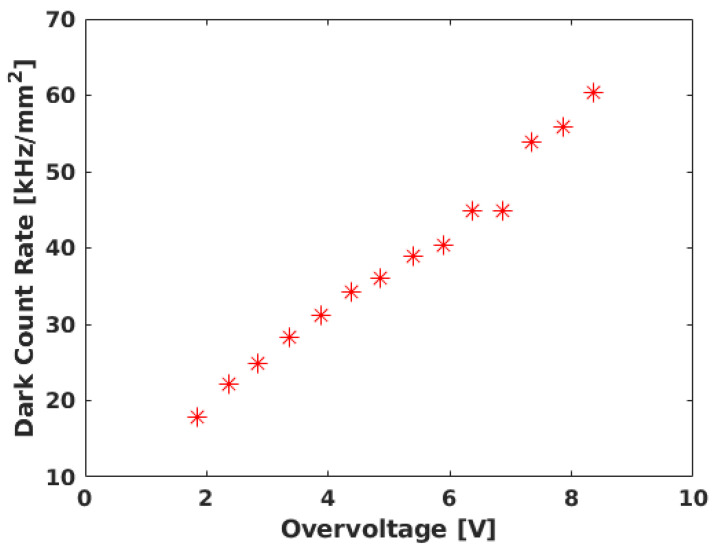
DCR for Hamamatsu SiPM S13360 for different values of overvoltage. Adapted from [22].

SiPMs could improve the performances of neutrino telescopes; nevertheless, they exhibit a notably high DCR, surpassing the capabilities of current neutrino telescope acquisition systems. Although the DCR has dropped from 1 MHz/mm^2^ to below 40 kHz/mm^2^ at ambient temperature in recent years, it remains substantially higher than in PMTs, posing a challenge for their integration in neutrino telescopes. For comparison, PMTs in KM3NeT have an average dark noise of about 0.28 Hz/mm^2^ [8], over five orders of magnitude lower than that of SiPMs. SiPM DCRs arise from thermal carrier generation, trap-assisted tunneling, or band-gap tunneling, resulting in a response equivalent to a single photon. The DCR is highly temperature dependent, halving its value every eight degrees [21]. Presently, state-of-the-art SiPMs exhibit a DCR of about 20–30 kHz/mm^2^ [22] at ambient temperature (see Figure 1), a challenge for the acquisition system that has thus far hindered their implementation in neutrino telescopes. At these noise rates, an SiPM with a detection area similar to that of a multi-PMT Digital Optical Module (DOM) [23] at seawater temperature would yield a DCR of approximately 30 MHz. This level of noise surpasses the capabilities of current readout systems, leading to communication bandwidth overload. While operating underwater, the temperature reduction would halve the SiPM DCR [24], and, in the case of ice, it reduces by about two orders of magnitude [25]. However, the noise level remains significant. Implementing a threshold above a single photoelectron could result in a 100-fold reduction in the DCR [6], albeit at the cost of losing single-photon event information. Therefore, it is imperative to incorporate a DCR rejection technique within the acquisition electronics.

## 4. Dark Count Rate Filtering

**Figure 2:**
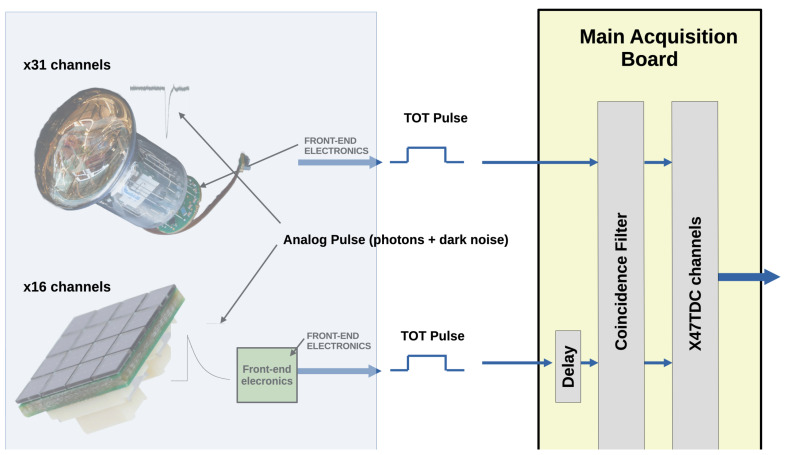
Possible layout for a hybrid DOM. The analog part, including the sensors, either PMTs or SiPMs, and the front-end electronics, is simulated with artificial pulses. The DCR filter and the TDC channels are implemented in ARTIX Field-Programmable Gate Arrays (FPGAs). The delay of the SiPM channels can be implemented in the routing of the ToT pulse to the FPGA, in the FPGA itself, or in both. In the figure, only the delay at the FPGA is shown, the approach taken in this work.

The state-of-the-art detection node in neutrino telescopes is composed of a glass sphere embedding several tens of Photomultipliers [23,26]. The acquisition front-end electronics of the first of these multi-PMT DOMs [27], developed for KM3NeT, converts the PMT analog pulses into a digital signal, which is active during the time the PMT signal is over a threshold, the Time over Threshold (ToT). The ToT signals are transmitted to a central acquisition board, where the main FPGA implements the TDCs. The TDCs timestamp the arrival time of the ToT pulses and measure the duration of the ToT. All the information acquired is sent on-shore for further analysis, the so-called “all-data-to-shore” approach. An improvement of the multi-PMT DOM involves the use of SiPMs together with a PMT. The main problem of using SiPMs is the high DCR, which saturates the bandwidth available. One potential approach in neutrino telescopes involves reducing the DCR by enhancing the “all-data-to-shore” scheme through the implementation of a coincidence filter between channels in the acquisition electronics and therefore not sending all the data but only the most relevant fraction. Figure 2 shows a simplified scheme of how the acquisition system could be with two acquisition channels, one with a PMT, acting as a trigger channel, and another with an SiPM. The analog pulses are digitized by the front-end electronics, converted into a ToT pulse, and sent to the acquisition board to be acquired by the TDCs, which timestamp the arrival time of the ToT pulse and measure its width. Using only SiPMs as triggering channels will not reduce the DCR significantly as coincidences of DCR pulses will not be filtered. An SiPM 1 MHz trigger signal, with a triggering window of 12 ns, implies about 1.2% or 0.12 MHz of coincidences or false positives with an SiPM channel with the same DCR. While the false positives are not a problem by themselves, as higher levels of trigger will reject them, the high rate saturates the bandwidth available, and therefore a hybrid DOM composed of PMTs for triggering SiPMs is the best approach for neutrino telescopes. The 31 PMTs of the KM3NeT multi-PMT DOM can be extended with several SiPMs (about 16) in the interstices of the PMTs. This implementation would lead to a higher detection area and higher directivity as the DOM will have more detection segments. In addition, the SiPMs provide better timing. In this hybrid DOM, a DCR filter could be used, using the PMT signals as triggers of the SiPM channels. The inclusion of a coincidence filter in the readout electronics is essential for reducing the substantial DCR produced by SiPMs and ensuring that the data sent to the central station remain within the available bandwidth. At the same time, all the PMT information is maintained. The drawback of this implementation is the case where the SiPMs detect photons but the triggering PMT does not as the SiPM signal will be filtered. Even if the efficiency of the SiPMs is higher than that of the PMTs, this case is expected to occur on rare occasions as the PMT detection area is significantly higher than that of SiPMs. The configurable time window for the filtering process, with a default value (12 ns) similar to the trigger window used in the KM3NeT DOM trigger, adds an element of adaptability as it is possible to adapt this window to the operation conditions. The processing of acquired data to perform coincidences within the acquisition electronics, which will effectively reduce the data rate destined for the central station, is one of the pivotal factors in reducing the DCR and therefore being able to use SiPMs for enhancing the angular resolution of neutrino telescopes. The incorporation of FPGAs would confer extra flexibility, allowing for remote upgrades of the DCR filter even with the detection nodes already deployed. It is important to embed the TDCs and the DCR filter in the same device as fast communication between them is mandatory for the filtering of the DCR.

## 5. Implementation

The implementation of the TDCs and the DCR filter are discussed in this section, together with the testbench where the validation of the DCR filter is performed.

### 5.1. Time-to-Digital Converter Implementation

To implement and test the DCR filter, a 1 ns TDC is developed. The TDC utilizes a multi-phase shift clock to minimize resource consumption, a critical consideration in applications such as neutrino telescopes given their operating conditions. Four phases of 250 MHz, each shifted by 90°, are employed. The transition of clock domains occurs in four steps of flip-flop chains, instead of the three used in other implementations, to guarantee the absence of timing issues. The multi-phase configuration is achieved by sampling the input with the primary clocks of both 250 MHz and their 90°-shifted counterparts generated by a phase-locked loop (PLL) along with their respective inverted clocks generated by NOT gates. By integrating these four clocks as the sampling clock in the flip-flop chains, the time domain shifts occur in 90° intervals (equivalent to one quarter of the TDC clock frequency), thus ensuring a constant input to meet the hold and setup time requirements of the flip-flops. The implemented TDC also allows for easy integration with the DCR filter.

### 5.2. Dark Count Rate Filter Implementation

**Figure 3:**
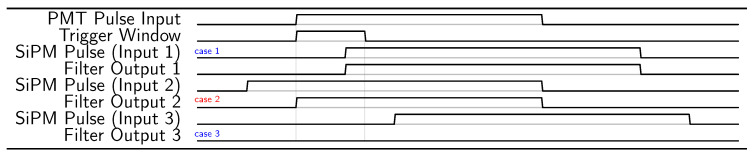
Timeline of the DCR filter behavior. Three different cases are shown, cases one and three in blue and case two in red. The trigger of the DCR filter is given by the PMT Pulse Input, which opens a trigger window of 12 ns. If an SiPM pulse occurs during the trigger window (case 1), the output of the filter is the same pulse. If the SiPM pulse occurs just before the opening of the trigger window (case 2), then the output of the pulse is the part of the pulse just after the opening of the trigger window. And finally, if the SiPM pulse is outside the trigger window, then the SiPM pulse is completely rejected.

The use of SiPMs together with PMTs will consume additional power. On the other hand, the power consumption of the sensors, which is about one seventh of the total KM3NeT DOM power consumption [27], can be totally, or at least partially, compensated by the use of a state-of-the-art FPGA. Still, the design of the acquisition electronics should reduce the use of resources. This is one of the main reasons why the filter of the DCR should be implemented using very few resources. The acquisition of all the events and posterior filter requires significant resources. It requires a huge buffer, and the events need to be ordered, which requires a significant use of computation resources. Both requirements are not available in neutrino acquisition systems, so a lower-resource architecture is needed. While the DCR filter reduces the bandwidth needed, the resource usage should be kept as low as possible, which limits the complexity of the filter. The filtering should be carried out when the data are ordered, and this only happens when they arrive. This is the approach taken in this work. The SiPM signals are rejected by default, and, only when a PMT signal occurs, which acts as a trigger, is the acquisition of SiPMs allowed. As the acquisition of all the channels is in the same FPGA, there are no timing alignment problems. The behavior of the DCR filter is exemplified in the timeline of Figure 3. The input from the slave TDC is intentionally delayed by 2.5 ns to anticipate the arrival of the PMT signal, which is detected by the master TDC. This delay can be implemented either in the FPGA (as in the present case), in the transmission line from the SiPM to the acquisition electronics, or with a combination of the two previous cases. A trigger window of 12 ns is initiated by a rise in the master input signal. If an event is detected in the slave TDC during this short window (i.e., light arrives at the SiPM), it is read out. Events starting before the trigger window is open and active during the trigger window are acquired from when the trigger window opens to the end of the input pulse. Finally, slave TDC events occurring outside of this 12 ns window are rejected and are not acquired. The Verilog code of the filter is presented in Listing 1.

**Listing 1.** Verilog implementation of the DCR filter testbench. If an event enters during the 12 ns window, the full event is acquired.

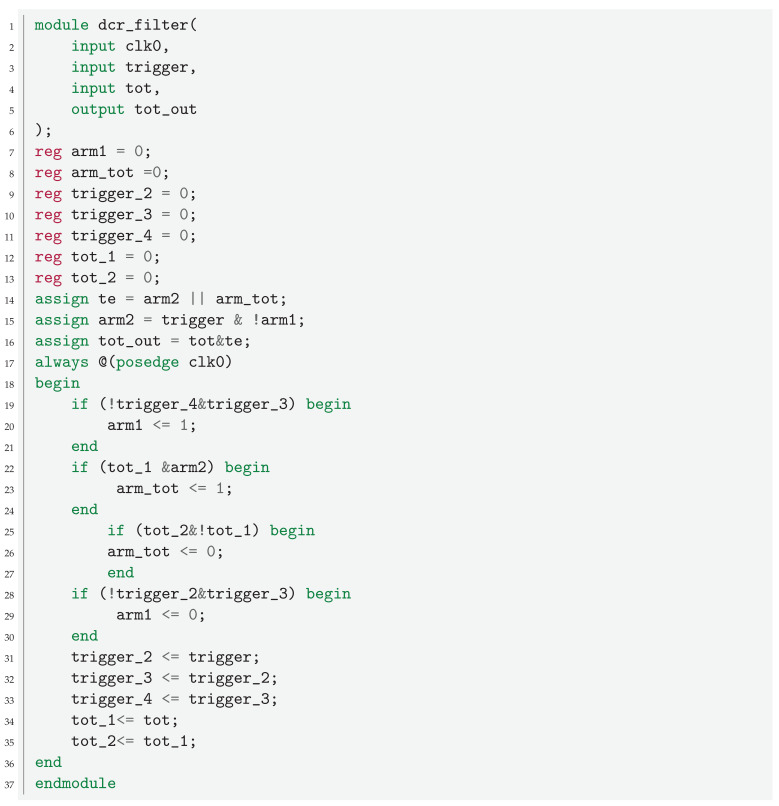



### 5.3. Testbench Implementation

The test is implemented in a Xilinx evaluation board, the Zedboard (https://digilent.com/reference/programmable-logic/zedboard/start, accessed on 3 February 2024), which has an ARTIX-7 (https://docs.xilinx.com/v/u/en-US/ds181_Artix_7_Data_Sheet, accessed on 3 February 2024) FPGA of 85k logic cells and a speed rate of −1. In order to test the DCR filter, two TDC channels are embedded in the FPGA. One of the TDC channels acts in master mode, which corresponds with the Photomultiplier Tube (PMT) channel, and one in slave mode, corresponding to the SiPM channel. The PMT acts as master as it triggers the acquisition windows in the slave channel, the SiPM TDC channel.

The TDC Intellectual Property core implements an Advanced Extensible Interface (AXI)-Stream slave, and the TDC data are transferred to the Double-Data-Rate Memory using a Direct Memory Access controller and two of the High-Performance AXI ports of the processor included in the FPGA. The delay of the slave TDC is implemented with the IODELAYE2 primitive (https://docs.xilinx.com/r/2021.1-English/ug953-vivado-7series-libraries/IDELAYE2, accessed on 3 February 2024). The DCR filter is located just before the SiPM TDC channel. The resources consumed by the DCR filter are just one slice lookup table and two slice registers by the DCR filter channel, with a negligible increase in the power consumption.

## 6. First Results

Two different tests are performed to validate the DCR filter proposed.

### 6.1. Test with Field-Programmable-Gate-Array-Generated Input Pulses

**Figure 4:**
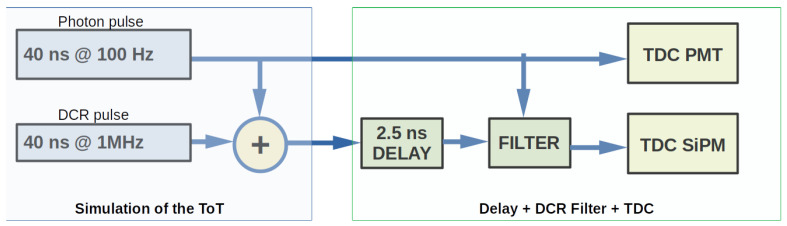
Architecture of the DCR filter testbench. The master TDC channel input acts as the trigger signal of the slave TDC channel. The trigger window of the slave TDC channel is only active during a short time (12 ns), initiated by the master TDC.

**Figure 5:**
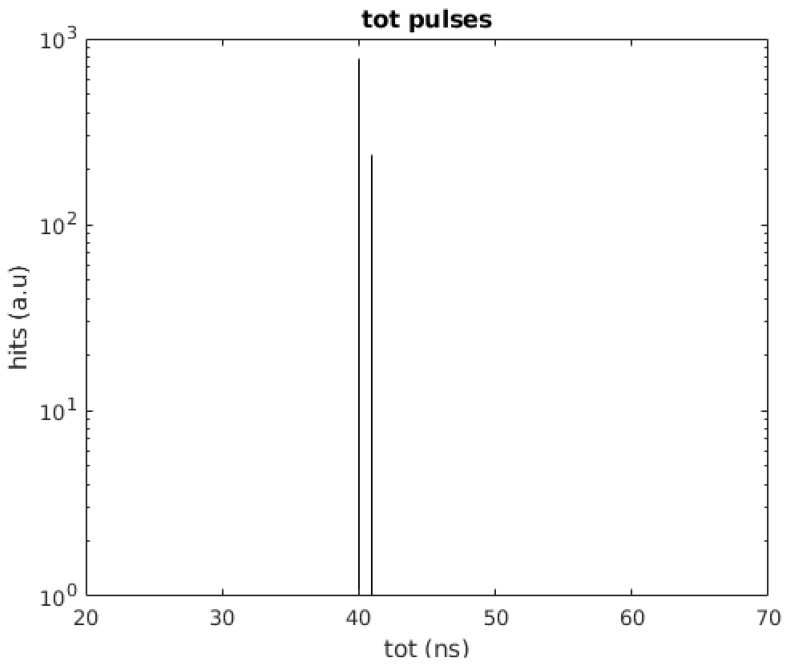
Time over Threshold obtained in the slave (SiPM) channel. The SiPM TDC is supplied with a pulse of 40 ns of width at 100 Hz (injected also in the PMT TDC channel and used as a trigger) and a simulated DCR of 40 ns of width at 1 MHz. The filter, triggered by the PMT TDC, successfully rejects the noise.

**Figure 6:**
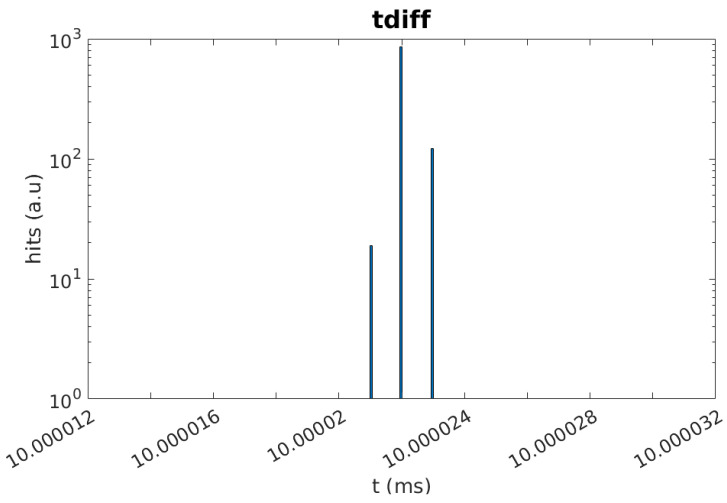
The difference between the arrival times of two consecutive events is shown in the figure. As can be seen, the distance between pulses is 10 ms, the frequency of the pulse injected in the PMT TDC, while the DCR pulses (40 ns at 1 MHz) are filtered.

In the first test, the PMT and the SiPM signals are generated internally in the FPGA. To test the DCR filter with realistic input signals, first, the master channel is supplied with a pulse with a width of 40 ns and a frequency of 100 Hz. The second channel is supplied with the same 40 ns pulse as well as a simulated DCR pulse with a width of 40 ns and a frequency of 1 MHz. Figure 4 shows the test scheme implemented. The 100 Hz pulse emulates the arrival of single photons at both the PMT and SiPM and is the pulse that should be read out. The selected pulse width is about 15 ns longer than the typical KM3NeT ToT for single photoelectrons, which is 25 ns, although it is still valid as the ToT depends on the front-end electronics and the chosen threshold. The same applies to the SiPM. The 1 MHz pulse simulates the DCR of the SiPM, which has a signature equal to the single photoelectron signal, and it is added to the SiPM photon signal before arrival at the TDC in a similar way as happens in an SiPM. The results obtained for these tests are shown in Figure 5 and Figure 6. The filter effectively removes the DCR pulses in the SiPM TDC channel while maintaining the light pulses. As can be seen, the time difference between consecutive pulses registered by the SiPM TDC channel is 10 ms, the frequency of the simulated light pulses.

### 6.2. Test with External Pulse Generator

**Figure 7:**
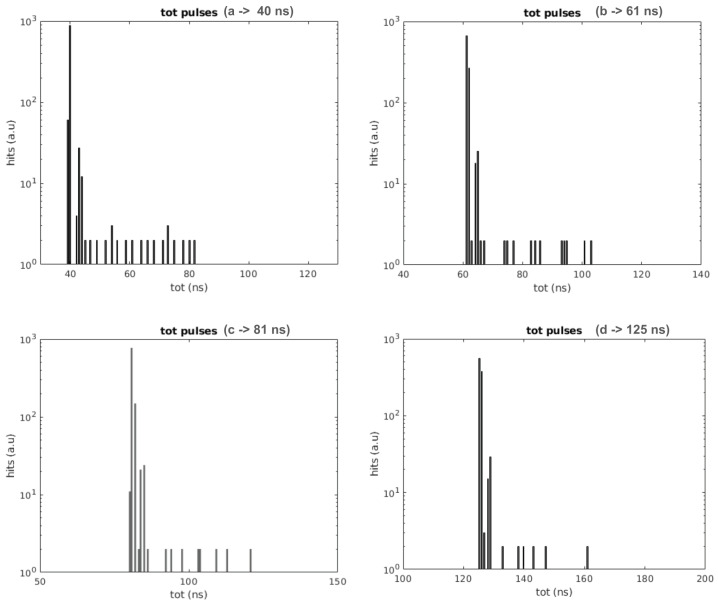
Results obtained when an external pulse generator is used to simulate the photon signals at the PMT (master) and SiPM (slave) TDCs. A 30 ns pulse DCR at 1 MHz is simulated with an internal FPGA pulse generator. Light pulse length is tested at 40 (**a**), 61 (**b**), 81 (**c**), and 125 ns (**d**). As can be observed, the DCR is, as expected, correctly filtered in all cases. There are some pulses with a longer duration with respect to the photon pulse. In these cases, the DCR adds to the photon signal, resulting in a longer ToT. The maximum ToT observed corresponds to the light ToT plus the DCR ToT pulse.

In the second test, the simulated light pulse is generated by an external pulse generator. The phase of the external pulse generator is not correlated with the phase of the FPGA clock so the coincidences between the simulated PMT signal and the SiPM signals are purely random. This is similar to the use of an external pulse generator to qualify the non-linearities of Time-to-Digital Converters using a statistical code density test [28,29]. An AWG.4022 from Active Technologies with an electrical rise time of 800 ps is used to that end. Four pulse widths are tested, one at 40 ns, as in the previous test, and the other three at 61, 81, and 125 ns, simulating signals generated by more than one photon. The results, which again show that the 30 ns DCR pulse is removed, are presented in Figure 7. In the four cases, it is possible to see that there are a few pulses with longer duration than the photon pulse. The DCR pulse adds, in some cases, to the photon pulse, extending the total duration of the pulse. As can be seen, the total duration does not exceed the photon pulse length plus the DCR pulse length. This is not observed in the internally generated photon pulse (see previous section) as they are correlated to the DCR pulses, and, in the case in which they superpose, they superpose completely. The addition of the DCR pulse can happen at the end or the beginning of the photon pulse. In the case in which it is added at the end of the photon pulse, there is an extension of the ToT, which can be extended up to the maximum possible, that is, the photon pulse plus the DCR pulse. In the case in which the DCR pulse is added at the beginning of the pulse, it happens as in case 2 of the timeline presented in Figure 3; the DCR part of the pulse is almost completely rejected, and only the photon pulse plus the delay of the DCR filter remains. The small accumulation of ToTs with a length of about 3 ns longer than the photon pulses corresponds to this effect. In almost all cases, with the exception of when the dark noise is coincident with the light pulses, the DCR pulses are rejected.

## 7. Conclusions

A filter architecture to reject the DCR of SiPMs installed in neutrino telescope nodes has been presented. PMT channels are used to trigger the SiPM channels, only allowing the readout of SiPM data during a small trigger window coincident with the detection of light by PMTs. The DCR filter includes a delay of the SiPM or slave channel to allow the trigger to arrive before an event in the slave channel appears. If an event arrives in the slave channel while the acquisition window is open, the full event is acquired. A proof of concept of the DCR filter with two TDC channels, one in slave mode and the other in master mode, has been implemented and tested, achieving an almost complete rejection of the simulated DCR. The present development paves the way for the use of SiPMs in neutrino telescopes, which would improve the angular resolution. Future work will include an increase in the number of channels up to 32 masters and 32 slaves and the use of different filter schemes.

## Data Availability

Data are contained within the article.
